# Development of a Novel Electrostatic-Based Bioaerosol Sampler

**DOI:** 10.3390/mi15091068

**Published:** 2024-08-24

**Authors:** Zirui Pang, Lulu Shi, Wei Liu, Wenru Liu, Xin Tian, Mingyu Wang, Jifang Tao

**Affiliations:** 1 Key Laboratory of Laser and Infrared System Ministry of Education, Shandong University, Qingdao 266237, China; pangzirui@mail.sdu.edu.cn; 2 State Key Laboratory of Microbial Technology, Shandong University, Qingdao 266237, China; shill0712@163.com (L.S.); wangmingyu@sdu.edu.cn (M.W.); 3 Qingdao Institute of Measurement Technology, Qingdao 266000, China; lwlizzie@163.com (W.L.); 18705151696@163.com (W.L.); 4 School of Physics and Electronic Information, Weifang University, Weifang 261061, China; tianxin1012@mail.sdu.edu.cn; 5 School of Information Science and Engineering, Shandong University, Qingdao 266237, China

**Keywords:** bioaerosol, electrostatic collection, corona charge, enrichment

## Abstract

On-site bioaerosol monitoring is essential for estimating microbial biomass and mitigating the risk of infection induced by aerosol transmission. This study introduces a novel electrostatic bioaerosol sampler, which is fabricated by the use of 3D printing, for rapid bioaerosol collection. Aerosol particles were charged and enriched in the sampler. Relationships between particle sizes and collection efficiencies under varying charging voltages were established using a charging model. The design of the sampler was optimized using commercial software, incorporating electrostatic field analysis, computational fluid dynamics (CFD), and particle trajectory simulations. To validate the sampler’s collection efficiency, polystyrene (PS) spheres in an aerosol dispenser were atomized into an aerosol. The sampler collection efficiency exceeded 90% for particles larger than 1.2 μm under an applied voltage of 4.7 kV and an airflow rate of 2 L/min. The enrichment capacity was greater than 153,000 for particles larger than 1.2 μm under an applied voltage of 4.7 kV and an airflow rate of 8 L/min. With the merits of low cost, miniaturization, and high collection efficiency, the sampler can be used to collect samples on-site and in remote areas to verify the pathogens and reduce the risk of infection through aerosol transmission.

## 1. Introduction

Aerosol transmission is one of the major modes of transmission of infectious respiratory particles [1]. SARS, influenza, tuberculosis, and the recent pandemic severe acute respiratory syndrome coronavirus 2 (SARS-CoV-2) can be transmitted via aerosols [2,3,4,5], and the survival time of SARS-CoV-2 is nearly 3 hours [6]. Thus, there is an urgent need for a novel bioaerosol sampler for the rapid collection of bioaerosols, enabling the effective collection and detection of airborne particles and the monitoring of air quality.

Several methods have been developed for the collection of bioaerosols, such as the impingement [7], filtration [8], condensation particle growth [9,10], wet cyclone [11], microfluidic-based techniques [12,13], and the electrostatic sampler [14,15]. However, during the separation of microorganisms from airflow with impingement, such as with AGI-30 and BioSampler, microorganisms are inactivated by the shear force [7] and the evaporation of liquid collection medium [15]. Microorganisms captured in the filter membrane can be inactivated not only during the evaporation of the buffer with filtration, but the captured species of microorganism is also limited by the hole size of the filter membrane [8]. Smaller-sized microorganisms in nano scale can be captured and counted with condensation particle growth, whereas the most-used solutions for cooling saturated vapor, such as butanol and isopropanol, are flammable and harmful to human body [9,10]. The wet cyclone method is not suitable for the collection of bioaerosols at low temperature areas as a result of using a liquid buffer [11]. Ensuring the passage of an effective amount of air through the microchannels [12] and optimizing the collection process for large sampling volumes in a reduced time frame [16] continue to be significant challenges in microfluidic-based techniques.

The electrostatic-based technologies have been used in various applications such miniaturized integrated equipment [17,18]. The principle of electrostatic bioaerosol sampler is similar to the electrostatic precipitation and has been used for the collection of bioaerosols including those of virus and bacteria [7,14,15,19]. Airborne particles are charged in the charging device and captured in the collection aera under an electric field. As a result of high effectiveness and a high range of target particles, an electrostatic sampler is used for the collection of bioaerosol containing pathogenic microorganisms. Combining the advantages of electrostatic collection, here, we developed a 3D-printing-based bioaerosol sampler. 3D printing technology has been instrumental in integrating the essential components into the sampler design, resulting in a compact and efficient device that is suitable for bioaerosol collection in remote areas and special environments. Combined with colony culture, molecular diagnosis, and other detection and analysis methods [7,14,15,20], it provides a new method for the collection of pathogenic microorganisms in densely populated and high-exposure-risk places, such as schools, stations, and hospitals.

## 2. Materials and Methods

**Figure 1:**
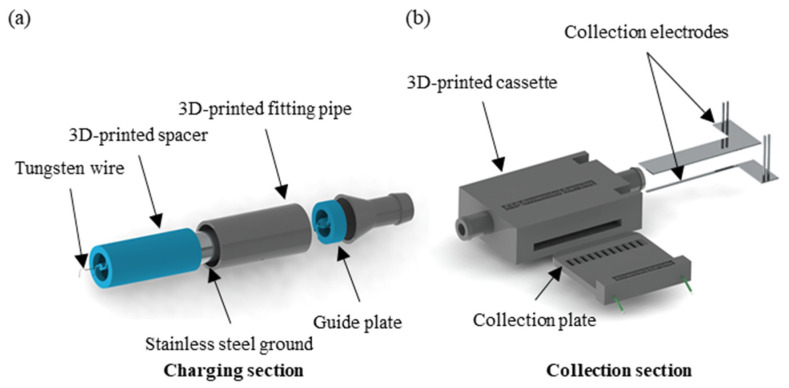
Enlarged view of the electrostatic bioaerosol sampler. Schematic illustration of charging section (**a**) and collection section (**b**).

The schematic illustration of the electrostatic bioaerosol sampler is shown in Figure 1. The device contains a charge section and collection section. The charger section consists of tungsten wire with a 50 μm diameter, a stainless-steel ground electrode with an 8 mm inner diameter, a guide plate, a 3D-printed spacer, and a fitting pipe as depicted in Figure 1a. A non-uniform electric field is established between the tungsten wire and the stainless-steel ground electrode, which facilitates the corona discharge. This discharge serves to charge particles across the entire region, enhancing the collection process. The collection section comprises electrodes, a collection plate, and a cassette as depicted in Figure 1b. Upon passing through the charging section, the air near the discharging electrode experiences a dielectric breakdown, which leads to ionization. This ionization causes the electrons to be accelerated to the point where they can collide with other air molecules, thereby stripping the electrons and initiating a process of electron multiplication. This results in the formation of a dense cloud of positive ions and free electrons around the discharging electrode [21]; the aerosol particles are charged when they pass through this charging section. Particles are captured in the collection plate under the influence of the electric field generated between the electrodes in the collection section.

### 2.1. Calculation and Optimization

As a result of the low electrical charge carried by the microorganisms, bioaerosols experience negligible electric force and electric mobility under a uniform electric field. This is insufficient for effective electrostatic collection. Thus, a charging process is essential to enhance the electric charge of bioaerosols prior to collection. Bioaerosols become charged as they traverse a dense cloud of positive ions and free electrons, which is generated around the discharging electrode under the application of a high voltage during the charge collection process [21].

The total charge of particle carried after passing the charging section can be expressed as follows:

(1) $$q=\pi \varepsilon_{0}E_{m}d_{p}^{2}[(1+\frac{2\lambda }{d_{p}}{)}^{2}+(\frac{2}{1+\frac{2\lambda }{d_{p}}})(\frac{\varepsilon_{r}-1}{\varepsilon_{r}+2})]$$

where $\varepsilon_{0}$, $E_{m}$, $d_{p}$, λ, and $\varepsilon_{r}$ are the vacuum permittivity, electric field, particle diameter, mean free path, and particle dielectric constant, respectively [22].

In the analysis of the forces acting on the bioaerosol particles, they were approximated as rigid spherical pellets. The primary forces influencing the aerosol particles within the collection section include the electric field force, aerodynamic drag, gravitational force, and buoyant force. Compared to the electric field force, gravitational force and buoyant force are negligible, being approximately 3 to 6 orders of magnitude smaller. Therefore, our analysis primary focuses on the influence of the electric field force and the aerodynamic drag on the particles.

The electrostatic collection effectiveness can be expressed as follows:

(2) $$\eta =1-exp(-\frac{A}{Q}V)$$

where *A* and *Q* are the area of collection plate and sample flow rate, respectively [23]. *V* is the velocity of the particle under an electric field and can be expressed as follows:

(3) $$V=\frac{qC_{c}E_{m}}{3\pi \mu d_{p}}$$

where $C_{c}$ and μ are the Cunningham correction factor and aerodynamic viscosity, respectively [23].

The amount of charge for various particle size ranges can be calculated with theoretical modules. The design of the bioaerosol sampler is based on the velocity and electric mobility of the particles. To gain a comprehensive understanding of the sampler’s structure and to optimize its performance, we conducted a series of simulations. Specifically, to investigate the influence of airflow velocity distribution and particle dynamics within the charge section on the sampler’s collection efficiency under operational conditions, computational fluid dynamics (CFD), electrostatic analysis, and particle trajectory simulations were performed. Additionally, CFD simulations were performed to optimize the collection section’s structure, assessing the performances both before and after optimization. All simulations were conducted using the COMSOL Multiphysics software, following the establishment of the charger structure’s dimensions using SolidWorks.

It was reported that the atmospheric concentration of the COVID-19 virus was only 10^3^ to 10^4^ copies/m^3^ within the negative pressure isolation room that the patient living in [24]. It is critical to enrich the bioaerosols to address the challenge of detecting low concentrations of microbes in the environment. Given that the limit of detection (LOD) for a quantitative polymerase chain reaction (qPCR), which serves as the gold standard for nucleic acid amplification, is 10^3^ copies/mL [25], an enrichment factor of at least 10^5^ would be required to enhance the detection. To achieve this enrichment, an aerosol to hydrosol (ATH) method was selected for its simplicity and effectiveness in concentrating microorganisms. This method involves the transfer of microorganisms from the aerosol phase to a collection buffer, and it can be integrated with the electrostatic bioaerosol collectors. However, the airflow carrying the aerosols across the collection buffer not only expedited the evaporation of the buffer but also led to a buffer overflow from the collection plate. To mitigate these effects, microbridges were incorporated above the collection buffer which isolated the buffer from airflow while retaining the opening windows to facilitate the entry of bioaerosols into the collector. Simulations were carried out to have a better understanding of the performance of those microbridges.

### 2.2. Fabrication of the Bioaerosol Sampler

**Figure 2:**
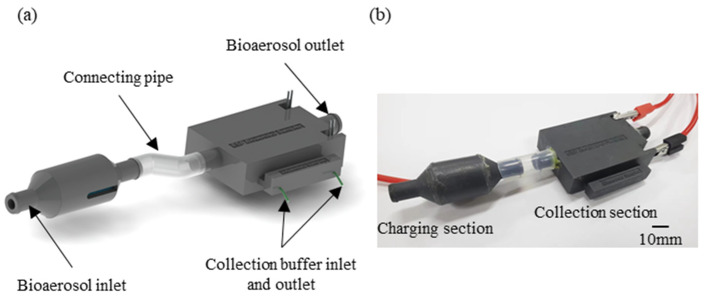
Schematic illustration (**a**) and image (**b**) of the electrostatic bioaerosol sampler.

The schematic illustration of the electrostatic bioaerosol sampler is presented in Figure 2a. The body of the bioaerosol sampler was designed with SolidWorks and fabricated via 3D printing. Using 3D printing technology provides us with a cost-effective and adaptable manufacturing approach for a bioaerosol sampler that is suitable for use in a resource-limited area. Insulating resin was selected for the fabrication of the sampler to enhance the performance when operating at high voltages. During assembly, the charge section was integrated with a tungsten wire and a stainless-steel ground electrode, while the collection section was integrated with electrodes and a collection plate. This assembly was sealed using resin glue to prevent any leakage of the aerosol samples. Those two sections were connected by a silicone hose as depicted in Figure 2b.

### 2.3. Evaluation of the Bioaerosol Sampler

**Figure 3:**
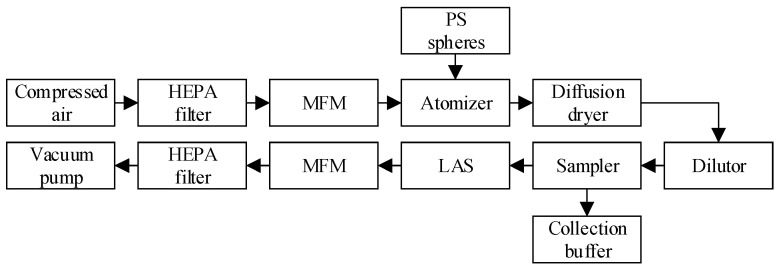
Experimental setup for aerosol sampler test.

To evaluate the performance of the bioaerosol sampler, we established an experimental setup depicted in Figure 3. Initially, compressed air was passed through a high efficiency particulate air (HEPA) filter to ensure purity before being used to aerosolize the polystyrene (PS) microspheres in an aerosol dispenser, with the flow rate being controlled by a mass flow meter (MFM). The resulting aerosolized PS microspheres were then directed into a diffusion dryer to minimize the impact of water vapor on the sampler performance. Following mixing and dilution in a dilutor, aerosolized PS spheres were introduced into the sampler. While one fraction of the microspheres was captured into the buffer solution for subsequent analysis, another fraction was directed to a laser aerosol spectrometer (LAS) to determine the particle size distribution and concentration. The LAS readings were obtained by activating and deactivating the sampler control, which facilitated the calculation of the collection efficiency of the sampler. Finally, the aerosol samples were drawn through a vacuum system, regulated by a flow controller, and filtered through a HEPA filter.

PS spheres were employed to test and evaluate the performance of the designed electrostatic sampler as its density and charge number were similar to that of bioaerosol particles [15,26,27]. Those PS spheres were diluted into the deionized (DI) water and ultrasonic agitation was applied to minimize the adhesion. According to the operating principle of the electrostatic bioaerosol sampler, the collection efficiency is primarily influenced by the velocity of airflow and charging voltages. Consequently, we investigated the performance of the sampler across a range of flow rates and charging voltages to gain a comprehensive understanding of its behavior under varying conditions. The collection efficiency can be expressed as follows:

(4) $$\eta =1-\frac{C_{on}}{C_{off}}$$

where $C_{on}$, $C_{off}$ are the amounts of PS spheres counted by LAS with the sampler switched on or off, respectively. The enrichment ${EC}_{ATH}$ can be expressed by the following equation:

(5) $${EC}_{ATH}=\frac{Q_{A}\times \eta }{Q_{L}}$$

where $Q_{A}$, $Q_{L}$ are the flow rates of the airflow and buffer, respectively.

### 2.4. Testing Methods

The electrostatic bioaerosol sampler was integrated into the aerosol experimental setup, positioned downstream of an aerosol dispenser, diffusion dryer, and dilutor. The diffusion dryer was incorporated to minimize the influence of humidity on the sampler performance. An LAS instrument was placed immediately downstream of the sampler to monitor particle size distribution. The gas tightness of the entire workflow was confirmed to ensure no leaks, and the airflow rate was calibrated using an MFM.

During the experimental procedure, the particle size distribution was measured by the LAS, both when the sampler was activated and when it was switched off, to assess the sampler’s collection efficiency. The LAS was set to measure particle concentrations at intervals of 10 s, with a total collection time of 120 s per measurement cycle.

## 3. Results and Discussion

**Figure 4:**
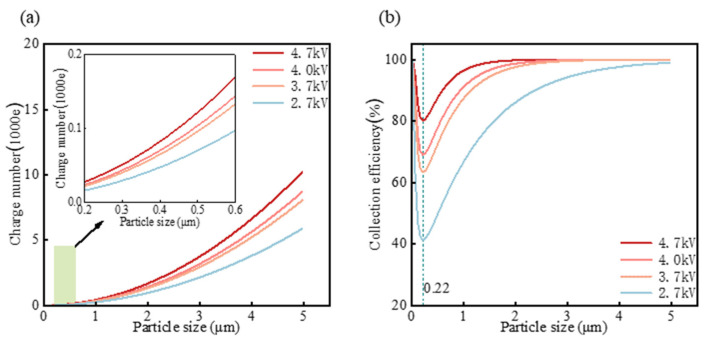
Calculation results of charge number distribution (**a**) and collection efficiency (**b**) based on calculation model. The insert in (**a**) shows a magnified view of the green area.

Electrical charges carried by the particles within the size range of 0.1 to 5 μm were calculated using the Cochet mode, and the results are presented in Figure 4a. Figure 4a illustrates that electrical charges increase with both particle size and charging voltage. However, the collection efficiency exhibits a distinct trend where it decreases with the increasing particle size, from 0.1 to 0.22 μm, likely due to a decrease in the electric mobility in this size range. Conversely, collection efficiency rises from 0.22 to 5 μm, reaching nearly 100% with the particle size at 5 μm. Furthermore, collection efficiency increases with higher charging voltage. These calculation findings indicate that the electrostatic bioaerosol sampler not only exhibits high collection efficiency for larger particles but is also effective for submicron particles, including bacteria and viruses.

It is important to note that additional culturing and analysis are required to fully assess the species composition and quantity of the microorganisms captured, as the integrity of the sample is crucial for practical applications. While the collection efficiency of the sampler increases with the application of higher charging voltages, the corona discharge process generates ozone, which can induce reactive oxygen species (ROS), such as H_2_O_2_ and ^1^O_2_, and the electrophoretic deposition at high voltages may cause membrane deformation, potentially leading to the inactivation of microorganisms [28,29,30]. Consequently, selecting an appropriate collection voltage is essential to maintain the integrity of the microorganisms or biomarkers within the sample.

**Figure 5:**
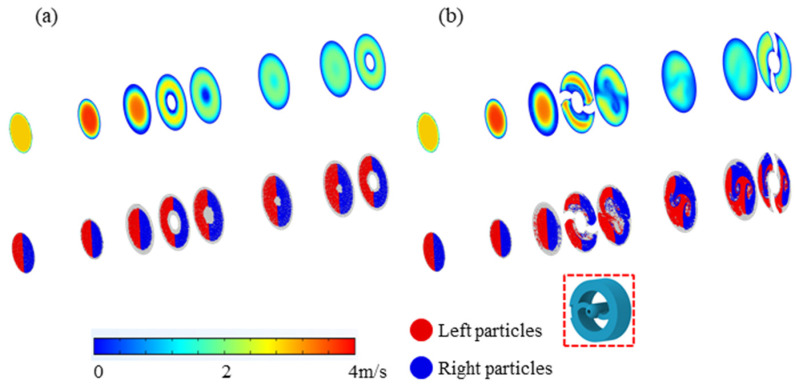
Simulation of CFD and particle trajectory before (**a**) and after (**b**) optimization with the guide plate, with the guide plate highlighted by a red dotted line.

The results of the computational fluid dynamics (CFD) and particle trajectory simulations are depicted in Figure 5. The upper graph in Figure 5a reveals that the flow velocity decreases progressively from the center of the inlet towards the side walls, with velocity approaching zero near the sampler walls. This phenomenon is primarily attributed to the laminar flow characteristics, where the gas flow near the walls experiences increased viscous effects, resulting in reduced velocity due to friction between the stationary fluid and the moving fluid.

In order to mitigate the impact of the boundary layer on the aerosol particle motion within the sampler, a guide plate was strategically designed and placed at both the inlet and outlet. The velocity distribution and particle state distribution under the influence of the deflector are illustrated in Figure 5b, respectively. Here, red and blue particles denote the initial positions on the left and right sides of the inlet. Subsequent simulations with the optimized structure demonstrated the changes in the airflow and particle pathways, effectively mitigating the boundary layer influence caused by the walls.

**Figure 6:**
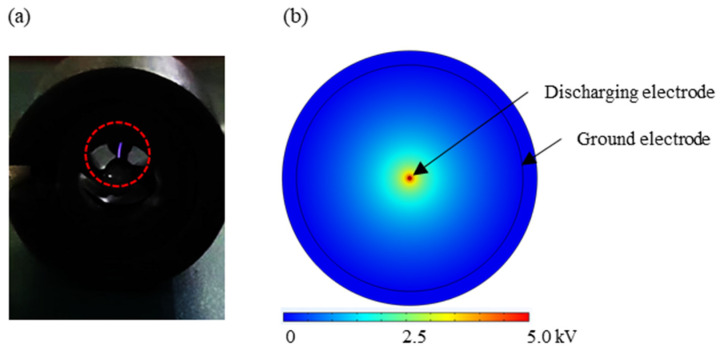
(**a**) Corona discharge glow observed during the discharging process highlighted by a red dotted circle. (**b**) Simulated electrostatic field distributions within the charging section of the sampler.

Figure 6a illustrates the discharging process, with the red dotted line delineating the glow produced by the corona discharge. Simultaneously, the electric field distribution at the discharging voltage was computationally modeled. As depicted in Figure 6b, a highly non-uniform electric field was observed between the discharging electrode and the ground electrode. The simulations’ results were experimentally validated when we observed the corona discharge glow during our trials, confirming the effectiveness of the charging section. As the discharging voltage increased, the air within the charger experienced a dielectric breakdown. To mitigate the impact of the charging voltage on the bioaerosols and to ensure the stability of the corona discharge, we established a maximum charging voltage threshold of 4.7 kV. The detailed study of the electrostatic field was essential for ensuring the reproducibility and reliability of the sampler’s performance.

**Figure 7:**
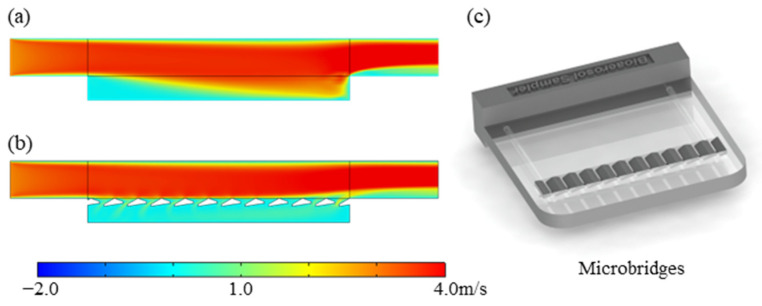
Simulation of CFD before (**a**) and after (**b**) optimization of the collection plate. (**c**) Schematic illustration of the collection plate incorporating microbridges.

We also investigated the design modifications intended to mitigate the impact of aerosol flow on the collection buffer through computational fluid dynamics (CFD) simulations. The results of these simulations, both before and after optimization, and the microbridges are presented in Figure 7. Figure 7a shows the pre-optimization flow velocity distribution. Notably, the simulation revealed the presence of negative flow velocity and the formation of a vortex within the collection plate, indicating that the flow direction here is opposite to that at the gas inlet. This reversed flow could potentially lead to buffer overflow within the collection plate, highlighting a design inefficiency that necessitated optimization.

To mitigate the influence of airflow on the buffer in the sampler, the collection plate was optimized by incorporating microbridges above the collection buffer as depicted in Figure 7c. Those microbridges were designed to isolate the buffer from airflow while retaining the opening windows to facilitate the entry of bioaerosols into the collector. The simulation results of the flow dynamics after optimization are shown in Figure 7b. Following optimization, a majority of the airflow was effectively isolated from the buffer area, with a significant reduction in the flow rate and the occurrence of secondary flows beneath the microbridges. These optimizations are crucial for maintaining the effectiveness of the collection system, ensuring accurate and reliable results during the capture and analysis of aerosol samples. Furthermore, by utilizing simulations prior to the experiments, research cost and duration of experimental process were significantly decreased.

**Figure 8:**
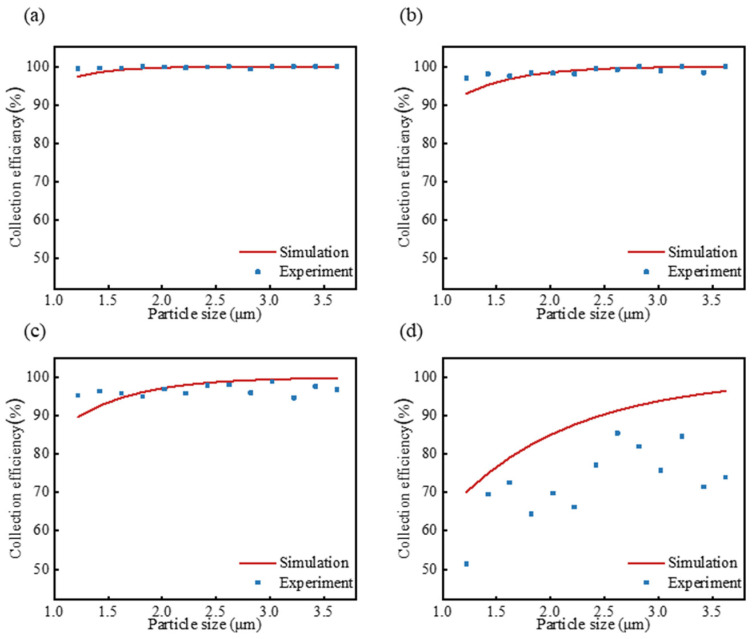
Comparison of experimental and calculated collection efficiencies for PS spheres at different applied voltages: (**a**) 4.7 kV, (**b**) 4.0 kV, (**c**) 3.7 kV and (**d**) 2.7 kV. Experimental data are represented by points, while calculated values are shown with lines.

To validate the collection efficiency calculated using a theoretical formula, PS spheres were utilized in the experiments conducted, at an airflow rate of 2 L/min and charging voltages of 4.7 kV, 4.0 kV, 3.7 kV, and 2.7 kV, as shown in Figure 8. The collection efficiency increased with the rise in collection voltage, which aligns with the simulation results. Moreover, the increase in collection efficiency can be attributed primarily to an enhancement in the charge number and electric mobility of the particles. Conversely, the collection efficiency decreased as the airflow rate increased, likely due to the high velocity reducing the collection time and diminishing electric mobility. The experimental collection efficiency for PS spheres smaller than 1.5 μm was found to be lower than the calculated efficiency at the charging voltages of 4.7 kV, 4.0 V, and 3.7 kV, as depicted in Figure 8. The results correspond with those of the previous studies [15,26]. This discrepancy is attributed to the fact that, during operation, both the charging section and the collection plate captured PS spheres, which led to an increase in the efficiency calculated. In contrast, the calculated collection efficiency exceeded the experimental efficiency at the 2.7 kV charging voltage. This was because the electric field at this voltage was near the critical value for corona discharge, which resulted in the insufficient charging of the sample, and therefore the PS spheres were not fully captured by the collection plate [7]. Thus, it is important to choose a proper charging voltage to maintain high collection efficiency to collect aerosol samples of various sizes.

**Figure 9:**
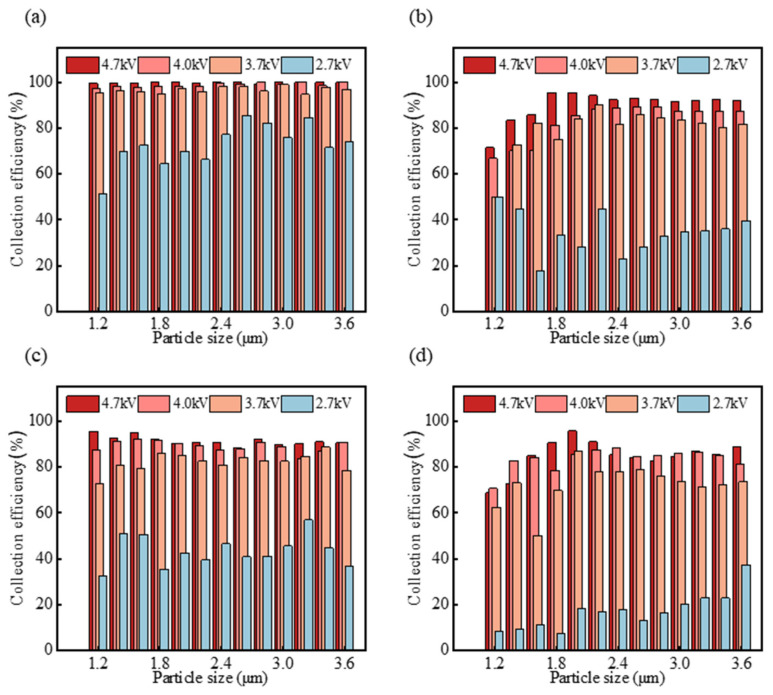
Experimental efficiencies of the sampler for PS spheres at different airflow rates: 2 L/min (**a**), 4 L/min (**b**), 6 L/min (**c**) and 8 L/min (**d**) with various applied voltages.

Furthermore, we assessed the efficiencies of the sampler with PS spheres at various charging voltages and flow rates, with the results presented in Figure 9. The collection efficiency exceeds 90% at a flow rate of 2 L/min and a charging voltage of 4.7 kV as depicted in Figure 9a. Figure 9c reveals that at a flow rate of 6 L/min and a charging voltage of 4.7 kV, the collection efficiency is over 80%. Furthermore, for PS spheres larger than 2 μm, the sampler achieves a collection efficiency of greater than 80% at flow rates of 4 L/min and 6 L/min, as shown in Figure 9b,c, with a charging voltage of 4.7 kV.

**Figure 10:**
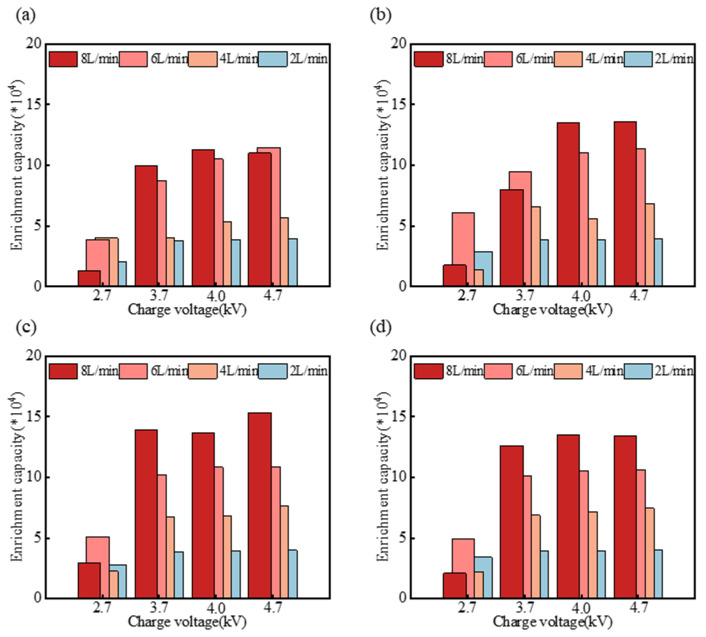
Enrichment capacity of the sampler for PS spheres with different diameters: 1.2 μm (**a**), 1.5 μm (**b**), 2.0 μm (**c**) and 2.5 μm (**d**) under various airflow rates and applied voltages.

We also calculated the enrichment of the sampler for the PS spheres collected in the buffer, and these results are depicted in Figure 10. The enrichment of the sampler reached maximum values of 114,700 for the 1.2 μm PS spheres, 135,800 for the 1.5 μm spheres, 153,100 for the 2.0 μm spheres, and 135,200 for the 2.5 μm spheres. These maximum enrichments were observed at an airflow rate of 8 L/min under 4.7 kV, and 6 L/min under 4.0 kV, respectively. This was because the elevated charging voltage enhanced the collection efficiency and positively influenced the enrichment capacity. The enrichment capacity was also simultaneously enhanced with the higher airflow rates [7,26,27]. These enrichment levels satisfy the concentration requirements for molecular diagnostics, including PCR [25].

## 4. Conclusions

In this work, we have developed a novel electrostatic bioaerosol sampler which has high efficiency in micron-scale particle collection with a low-cost and compact structure. Firstly, the charge number of the particles and the collection efficiency were calculated with theoretical models, which were critical for the design of the sampler. Then, CFD, particle trajectory analysis, and electric field distribution were simulated with commercial software to refine the structure of the sampler. The structure of the collection plate was optimized with microbridges to improve the airflow dynamics.

Our experimental validation confirmed that the collection efficiencies closely matched the simulation predictions. Specifically, the collection efficiencies exceeded 90% for PS spheres larger than 1 μm at an airflow rate of 2 L/min under an applied voltage of 4.7 kV. Furthermore, the enrichment reached 153,100 for particles with a diameter of 1.2 μm at an airflow rate of 8 L/min under 4.7 kV, which aligned with the requirements for molecular diagnosis techniques. However, due to constraints in the experimental conditions and throughput of the sampler, the capture of microorganisms in a real-world environment has not yet been demonstrated.

In the future, we aim to enhance the throughput and enrichment capacity with higher airflow rates to improve the detection capability for low concentrations of pathogens in aerosols. We also plan to integrate the sampler with a microfluidic chip that is equipped with PCR- or loop-mediated isothermal amplification (LAMP) technology. Collectively, our electrostatic bioaerosol sampler represents a low-cost, portable, and efficient solution. It has the potential to introduce a novel approach for bioaerosol collection, thereby contributing to a reduction in the risk of infection and interruption of transmission, particularly in remote areas.

## Data Availability

Data are contained within the article.
